# Therapeutic Effect of *Allium sativum* (Garlic) Extract Using Nanotechnology on Murine Chronic Toxoplasmosis

**DOI:** 10.1007/s11686-025-01142-8

**Published:** 2025-11-14

**Authors:** Doaa Abdulfttah Ahmad Amer, Fatma Mohamad El-Lessy, Ashraf M. Barakat, Rehab Mohamed El Shahat, Sabry A. Sadek, Reda M. Abdelhameed, Mona Mohammed Elderbawy

**Affiliations:** 1 Department of Parasitology, Faculty of Medicine, Al-Azhar University, Cairo, Egypt; 2 Department of Zoonotic Diseases, National Research Centre, Giza, Egypt; 3 Department of Pharmacology, Faculty of Medicine, Al-Azhar University, Cairo, Egypt; 4 Applied Organic Chemistry Department, National Research Centre, Chemical Industries Research Institute, Giza, Egypt

**Keywords:** Toxoplasmosis, Garlic, Nanoparticles, *Allium Sativum*

## Abstract

**Purpose:**

Current treatments for toxoplasmosis are often limited. This study aimed to assess the therapeutic efficacy of *Allium sativum* (garlic) extract loaded onto Fe-MOFs in a murine model of chronic toxoplasmosis.

**Methods:**

Sixty-five mice were assigned to seven groups. All groups, except the healthy control (GI), were infected with the *Toxoplasma gondii* ME49 strain. Treatments included Fe-MOFs (GIII), spiramycin (GIV), spiramycin@Fe-MOFs (GV), garlic extract (GVI), and garlic extract@Fe-MOFs (GVII). In vitro drug toxicity for garlic, Fe-MOFs, and garlic extract@Fe-MOFs were detected. Brain cysts counted, histopathological changes in various organs, and parasite DNA load (P29 gene) were assessed post-treatment using real-time PCR.

**Results:**

Spiramycin@Fe-MOFs (GV) and garlic extract @Fe-MOFs (GVII) groups showed a significant reduction in brain cyst burden (39.63% and 59.45%, respectively), along with marked improvement in histopathological changes compared to the other treated infected groups.

**Conclusion:**

These findings support garlic@Fe-MOFs as a potential treatment for toxoplasmosis, demonstrating enhanced efficacy, reduced toxicity, and improved histopathological outcomes.

## Introduction

*Toxoplasma gondii* (*T. gondii*) is an intracellular protozoan parasite that infects the global population and affects all warm-blooded animals [1]. Prevalence rates of *T. gondii* infection range from 10 to 90% worldwide [2], with marked variation between countries, within regions, and even across different communities in the same country based on cultural practices, hygiene, and dietary habits. Low seroprevalence (10–30%) has been reported in North America, Southeast Asia, Northern Europe, and Sahelian Africa, while moderate prevalence (30–50%) is observed in Central and Southern Europe. In contrast, the highest prevalence rates are found in Latin America and tropical African countries [3]. Notably, individuals aged 20–40 years are the most affected by toxoplasmosis [4].

It was first described in a North African rodent called the gondi. Infection occurs through ingestion of food or water contaminated with oocysts from cats or consumption of undercooked meat containing tissue cysts [5, 6].

*T. gondii* infection is often asymptomatic but may present with cervical lymphadenopathy, ocular involvement, or symptoms resembling mononucleosis, such as fever, sore throat, myalgia, rash, and, rarely, polymyositis or myocarditis. The risk of mother-to-fetus transmission increases later in pregnancy; however, early gestational infections tend to cause more severe complications, including intracranial calcifications and retinochoroiditis [7].

Immunocompromised individuals are at high risk for severe or life-threatening toxoplasmosis, which can result in neurological disorders and serious ocular complications, including blindness [7].

Diagnosing toxoplasmosis is challenging due to mild symptoms in healthy individuals and unreliable serology in immunosuppressed patients. ELISA is the main tool for detecting IgM and IgG antibodies [8]. PCR is valuable for detecting *T. gondii* DNA, especially in severe or CNS infections [9]. MRI is preferred for early lesion detection, and biopsy, though rarely performed, offers a definitive diagnosis by revealing tachyzoites and tissue cysts [8].

Treatment is recommended for immunocompetent individuals with severe or persistent symptoms and for all immunocompromised patients. The main goal is to control *T. gondii* replication during active infection. Prophylactic trimethoprim-sulfamethoxazole can help prevent acute infection in immunosuppressed individuals [10].

The standard treatment for *T. gondii* infection is pyrimethamine combined with sulfadiazine for 6 weeks, with dosing based on body weight and followed by maintenance therapy. Alternative drugs include clarithromycin, atovaquone, azithromycin, spiramycin, dapsone, and cotrimoxazole. Folic acid is co-administered to prevent deficiency caused by sulfadiazine [11].

Toxoplasmosis remains a major public health concern due to the limitations of current treatments, which can cause severe side effects like bone marrow suppression and hypersensitivity, and may lead to drug resistance. While existing therapies help control the acute phase, they are ineffective against encysted bradyzoites and congenital toxoplasmosis [12].

Research on *T. gondii* drug resistance has primarily focused on clarifying resistance mechanisms and drug action sites. Pyrimethamine resistance is associated with specific amino acid substitutions in parasite enzymes, while resistance to clindamycin, spiramycin, and azithromycin is linked to mutations in rRNA genes. In contrast, the mechanism underlying sulfadiazine resistance remains unclear. Notably, recent studies have reported a rise in sulfadiazine resistance; between 2013 and 2017, six resistant strains were identified, including TgCTBr11, Ck3, and Pg1, which were detected in both human toxoplasmosis cases and livestock intended for human consumption, these findings emphasize that ongoing drug resistance in *T. gondii* represents a growing challenge, underscoring the urgent need for more effective therapies [13].

Recent research has increasingly focused on the potential of herbal products in treating various diseases. Laboratory and experimental studies have demonstrated that certain plant extracts are effective against protozoan and helminthic parasites [14].

*Allium sativum* (garlic) is well known for its broad medicinal properties, including antibacterial, antiviral, antifungal, antiparasitic, antioxidant, and immunomodulatory effects [15, 16]. Its key bioactive compounds, allicin and sulfur-containing compounds, are responsible for these benefits. Studies have shown garlic extract to be effective against trematodes, cestodes, various protozoan parasites, and fungal organisms like yeast and Candida [17].

Garlic and its essential oil have strong antiparasitic effects against *T. gondii*, largely due to their anti-inflammatory and immune-modulating properties. Garlic shows promise as a natural treatment for toxoplasmosis and other parasitic infections by reducing pro-inflammatory cytokine levels [18]. Garlic-derived nanoparticles can enhance the efficacy of tumor immune checkpoint blockade therapy (cancer immunotherapy drugs) against solid gastrointestinal tumors when administered orally, showing greater effectiveness than isolated active compounds from garlic extract alone. These findings highlight the unique role of nanostructured bioactive substances in amplifying immune responses [19, 20].

Garlic extract can be effectively used to synthesize silver nanoparticles, improving their stability and functional efficiency. These biologically synthesized nanoparticles demonstrate significant antibacterial and antioxidant properties [21, 22].

Recent developments in nanomedicine have improved drug bioavailability, therapeutic efficacy, and targeted delivery [23, 24]. Metal-organic frameworks (MOFs) are promising materials used as templates for producing metal oxide nanoparticles with diverse and controlled morphologies [25].

MIL-101-NH2 is a popular iron-based metal-organic framework (Fe-MOF) used in medicine due to its stability, biocompatibility, tunable chemistry, and perceived safety, making it a strong candidate for drug delivery and other biomedical applications. Also, Fe-MOFs stand out owing to the low toxicity, high abundance, and redox properties of the iron element [26, 27]. The selection of iron (Fe) as the metal center in the MOF structure was based on its classification as a moderately toxic element, with relatively favorable biocompatibility when compared to other metals [28].

This study attempts to evaluate the therapeutic effectiveness of garlic extract@Fe-MOF nanoparticles in a murine model of chronic toxoplasmosis, hypothesizing that this Nano formulation would enhance drug delivery and reduce toxicity compared to the administration of free garlic extract.

## Materials and Methods

This study was conducted at the National Research Centre and the Faculty of Medicine, Al-Azhar University, between June 2023 and July 2024. All experimental procedures were performed in accordance with internationally recognized ethical guidelines. The research protocol was reviewed and approved by the Research Ethics Committee of the Faculty of Medicine, Al-Azhar University.

### Preparation of MIL-101-NH_2_

MIL-101-NH2 was synthesized as follows; 3.38 mmol ferric chloride hexahydrate (FeCl₃·6H₂O) and 5.5 mmol of 2-amino terephthalic acid were dissolved in a mixture of dimethyl formamide (DMF)/methanol (2:1 v/v) at room temperature. The obtained slurry was sealed and placed in the oven at 150 °C for 20 h. Finally, a light-brown product of MIL-101-NH2 powder was obtained. The product was filtered off and washed with DMF to remove the unreacted organic ligand and then washed again with methanol to exchange DMF. The final MIL-101-NH₂ powder was then dried under vacuum at room temperature [29, 30].

### Preparation of Garlic Extract Loaded MIL-101-NH_2_ (Fe) (Fe-MOF) Nanoparticles

To load the garlic into Fe–Metal-organic frameworks (Fe-MOFs) nanoparticles, ethanolic garlic extract with different concentrations (100–1000 ppm) was added to 1 g of Fe-MOF nanoparticles and stirred using a magnetic stirrer at 600 rpm for 90 min at room temperature. Then the solution was left undisturbed overnight. The suspension was then centrifuged at 5,000 rpm for 5 min and the supernatant and precipitate were separated. The amount of loaded drug was determined by finding the difference in garlic concentration in the solution before and after drug loading. The percentage of garlic loading was calculated using the following equation:

$$ {\mathrm{Percentage}}\;{\mathrm{of}}\;{\mathrm{garlic}}\;{\mathrm{loading}} = \left[ {\left( {{\mathrm{A}} - {\mathrm{B}}} \right)/{\mathrm{A}}} \right] \times 100 $$

where A represents the initial garlic concentration of the garlic solution while B represents the final garlic concentration [31, 32].

### Characterization of MOFs

The phase purity and crystallinity of prepared materials were characterized using X-ray diffraction (XRD) patterns (X’Pert MPD Philips diffractometer; the used monochromated was Cu Kα). The nanostructure morphology of MOFs was tested by scanning electron microscope (SEM) and transmission electron microscope (SEM: Hitachi SU-70, JP) [33, 34].

### Drug Toxicity

The cytotoxic effects of different concentrations of the newly synthesized nanocomposites (garlic, @Fe-MOFs, and Garlic@Fe-MOFs) were tested against the normal human epithelial cell line: BJ (normal skin fibroblast (NHDF, Sigma-Aldrich), using a multi-transaction translator (MTT) assay. The 48 h median lethal concentration values, LC50 and LC90, were estimated [35].

### Experimental Mice

A total of 65 laboratory-bred female Swiss albino mice, aged 6 to 7 weeks and weighing between 20 and 25 grams, were obtained from the Laboratory Animal House Unit at the National Research Centre. Mice were housed under standard laboratory conditions.

### Parasite

The *T. gondii* ME49 avirulent strain was obtained from the Zoonotic Diseases Department at the National Research Centre. The strain was maintained through regular serial passage in mice at the Zoonotic Laboratory to ensure a consistent supply of viable tissue cysts [36]. Cysts were harvested from the brain homogenates of sacrificed mice, diluted in 1 mL of sterile saline and prepared for use in the experimental infection of Swiss albino mice [37].

### Experimental Design and Infection

The mice were randomly divided into seven groups, ten mice in each group, except for the normal control group, which consisted of five mice. An infection with the *T. gondii* ME49 avirulent strain was induced orally using 20 cysts per mouse except in the normal control group [38].

Treatment commenced at 8 weeks post-infection and continued for 2 weeks. All mice were sacrificed by decapitation at 10 weeks post-infection for subsequent analyses. The animals were dissected, and brain samples were collected for both parasitological and histopathological examinations, while samples from the eyes, liver, spleen, and kidneys were obtained for histopathological analysis only [37].

### Experimental Groups and Treatments

The mice were divided into seventh groups as follows:

1. Group I (GI): Normal Control: Five healthy, non-infected, non-treated.
2. Group II (GII): Infected Control: Ten infected, non-treated.
3. Group III (GIII): Fe-MOF treatment: Ten infected, treated with Fe-MOFs at a dosage of 0.72 mg/kg, which was extrapolated from the in vitro LC₅₀ value (33.2 µg/mL) and adjusted for murine models in accordance with OECD Guideline 420 (Table 4).
4. Group IV (GIV): Spiramycin treatment: Ten infected, treated with spiramycin (tablets are crushed and dissolved into distilled water) at a dose of 100 mg/kg [37, 38].
5. Group V (GV): Spiramycin@Fe-MOFs treatment: Ten infected, treated with Spiramycin@ Fe-MOFs at a dose of 100 mg/kg [37].
6. Group VI (GVI): Garlic extract treatment: Ten infected, treated with garlic extract (powder dissolved into distilled water) at a dose of 200 mg/kg. This dosage was identified by Khoshzaban et al. [39] as the most effective, yielding the highest survival rate during the treatment of *T. gondii* infection with garlic.
7. Group VII (GVII): Garlic extract@ Fe-MOFs: Ten infected, treated with garlic extract@ Fe-MOF at a dose of 200 mg/kg [37].

Group V received the same dose as Group IV, and Group VII received the same dose as Group VI, in accordance with the protocol described by El Naggar et al. [33].

### Assessment of the Therapeutic Effects of the Studied Treatments

#### Parasitological sudy (brain cyst counting)

The brains of mice from each experimental group were harvested individually. Each brain was longitudinally bisected; one half was designated for parasitological evaluation of *T. gondii* cysts, and the other half was fixed in 10% neutral buffered formalin for histopathological Evaluation, as described by Al-Dakhil and Morsy [40] and El-Shafey et al. [41].

The extracted brains were homogenized in an equal volume of phosphate-buffered saline (PBS; pH 7.4) and passed ten times through a 16-gauge needle using a syringe to release tissue cysts, following the method of Dubey [42]. A glass slide was then coated with 50 µL (0.05 mL) of the brain homogenate. Cysts were counted microscopically. Brain cysts were quantified individually for each mouse, and the mean cyst count was subsequently calculated for each experimental group [43, 44].

#### Histopathological Examination

Specimens from the brain, eyes, liver, spleen, and kidneys of each mouse were fixed separately in 10% neutral buffered formalin. The tissues were then dehydrated through graded alcohol concentrations, cleared with xylene, and embedded in paraffin blocks. Sections of 5 µm thickness were prepared and stained with hematoxylin and eosin (H&E) to assess histopathological changes [37].

#### Molecular Identification

The liver tissue (20–25 mg) of the experimental animals was prepared for DNA extraction using a DNeasy Blood and Tissue Kit (Qiagen Co. Cat no. 69504, Germany) following the manufacturer’s instructions. Real-time polymerase chain reaction (RT-PCR) was conducted using 5X HOT FIREPol® SolisGreen® qPCR Mix (Cat. No. 5x 08-46-00001, Estonia) according to the manufacturer’s protocol.

The primer sequences were designed using Laser gene DNA star software V15. Then, 2 µL of the extracted DNA was mixed with 5 µL (5X) PCR master mix and 0.1 µL (50 nmol) of each designed forward and reverse primer targeting the P29 gene. The amplification of the targeted gene (P29) was performed for 10 min at 95 °C, followed by 40 cycles of 95 °C/10 s and 60 °C/20 s and 72 °C/20 s using a qTOWER3G Real-Time PCR System (AnalytikJena, Germany). The cycle threshold (CT) was determined when the fluorescence of a given sample significantly exceeded the baseline signal. A lower CT value represents a higher parasite load (DNA) and vice versa, while the negative CT represents the complete absence of the parasite. As shown in (Table 1).

**Table 1:** Sequences of P29 gene oligonucleotide primer used in RT-PCR assays

Primer	Sequences
P29 Q-f	CAGCATGGATAAGGCATCTG
P29 Q-r	GTTGCTCCTCTGTTAGTTCC

#### Statistical Analysis

Collected data were coded and introduced to a PC using the Statistical Package for Social Science (SPSS) for windows version 11.0. Data were represented as the mean ± standard deviation (SD) (n = 10). The analysis of variance (ANOVA) procedure was used to clarify statistically significant differences between the studied groups. Post hoc test (Bonferroni) for pairwise group comparison was used to assess inter group difference between each 2 groups. Values were considered statistically significant when *P* < 0.05. The infection reduction rate was assessed using the formula: (Mean value of the infected untreated group—mean value of infected treated group) × 100/Mean value of infected untreated group [43].

## Results

### Characterization of Nanoparticles

**Fig. 1:**
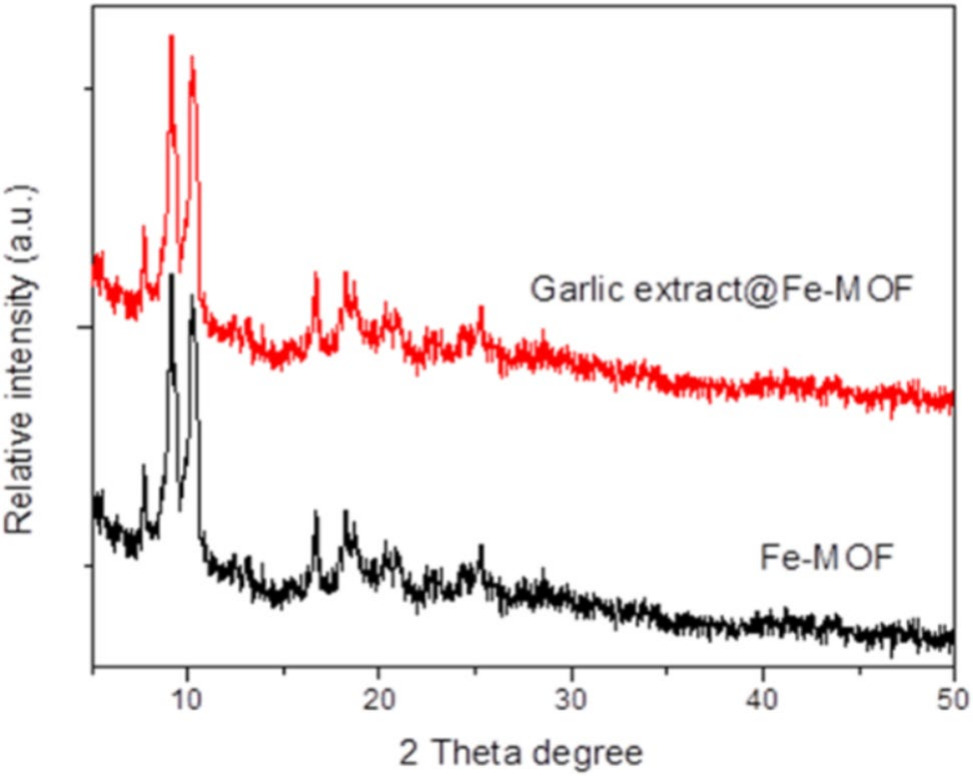
X-ray diffraction patterns of Fe-MOF [NH2-MIL-101(Fe)] before and after Garlic extract loading

Figure 1 Illustrates the Scanning Electron Microscopy (SEM) image of Fe-MOF [MIL-101-NH2(Fe)] both prior to and following the loading of garlic extracts [garlic extracts@Fe-MOF]. The PXRD pattern of Fe-MOF displays well-defined diffraction peaks at 2θ values of 9.1°, 10.2°, 16.6°, 18.2°, and 25.5°. The XRD pattern of the garlic extracts@Fe-MOF was in good accordance with the simulated Fe-MOF. Upon the addition of garlic extracts, which are distributed on the outer surface of Fe-MOF to create garlic extract@Fe-MOF, it is important to note that no nanoparticles are visible on the surface of Fe-MOF after the loading process. This observation indicates that the morphology of the original Fe-MOF material remains unchanged. Therefore, it can be concluded that the addition of garlic extract does not alter the morphology of Fe-MOF; however, a chemical bond may form between the garlic extract and Fe-MOF.

**Fig. 2:**
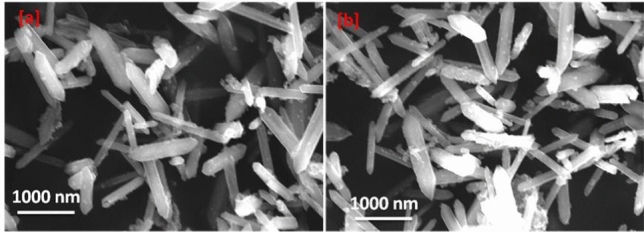
Micrographs for the Fe-MOF; **a** before Garlic extract loading), **b** after garlic extract loading

SEM images presented in (Fig. 2) illustrate the morphological characteristics of Fe-MOF before in panel [a] and after Garlic extract loading in panel [b]. The images reveal a distinct arrangement of nonuniform hexagonal rods structures.

### Garlic Extract Loading

The garlic extract loading efficiency of Fe-MOF nanoparticles have been examined. It depends on the concentration of garlic extract and the ratio of MIL-101-NH2 NPs. The garlic extract loading percentage increases with an increase in these parameters. It increases and becomes constant at a particular level. The loading amount is equal to 108.5 mg/g. as shown in (Fig. 2).

### Results of in vitro Bioassay of the Drugs

The sample was tested against the normal human dermal fibroblast (NHDF, Sigma-Aldrich) cell line BJ1. The sample was tested in a concentration range between 100 and 0.78 µg/ml using MTT assays. LC50 of Garlic alone, @Fe-MOFs and garlic extract@Fe-MOFs were 37.4%, 33.2% and 0% μg/ml, respectively. LC90 of garlic alone, @Fe-MOFs and Garlic extract@Fe-MOF were 70.8%, 69.5% and 0 % μg/ml, respectively. garlic extract@Fe-MOFs at 100 μg/ml increased the growth of the tested cells in 48 h by 44.7%. It had no cytotoxic effect in vitro (Table 2).

**Table 2:** Bioassay of the drugs loaded on Fe-MOFs in vitro

Sample	LC50 [ug/ml]	LC 90 [ug/ml]	Percentage of mortality of cells at drug concentration 100ug/ml
Garlic	37.4	70.8	95%
@ Fe-MOF	33.2	69.5	95%
Garlic extract @Fe-MOF	–	–	Growth of tested cells increased by 44.7%
Negative control	–	–	0%

### Parasitological Results

#### Brain Cyst Count

**Fig. 3:**
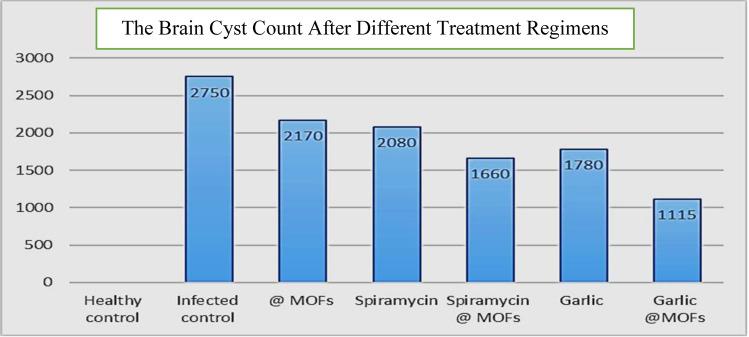
The brain cyst counts after different treatment regimens

As appeared in (Table 3) and (Fig. 3), the comparison of mean brain cyst counts across different treatment groups revealed that the infected control group (GII) exhibited the highest mean cyst count (2750 ± 29.4). All treatment groups showed statistically significant reductions in cyst counts compared to the infected control (*p* < 0.001). The most pronounced reduction was observed in the garlic extract@Fe-MOFs group (GVII), which recorded the lowest mean cyst count (1115 ± 21.8).

**Table 3:** Comparison of mean brain cyst counts among experimental groups

Animal group	Total no	No. of cysts		Pairwise group significance					
		Mean	SD	*P* against other groups					
				GII	GIII	GIV	GV	GVI	GVII
GII–infected control (a)	5	2750	29.4		b	c	d	e	f
				–	< 0.001	< 0.001	< 0.001	< 0.001	< 0.001
GIII–Fe-MOFs (b)	10	2170	27.4	A		c	d	e	f
				< 0.001	–	0.007	< 0.001	< 0.001	< 0.001
GIV–spiramycin (c)	10	2080	28.1	A	b				f
				< 0.001	0.007	–	0.054	0.519*	< 0.001
GV–spiramycin @ Fe-MOFs (d)	10	1660	28.2	A	b				f
				< 0.001	< 0.001	0.054	–	0.184*	0.002
GVI–garlic extract (e)	10	1780	25.7	A	b				f
				< 0.001	< 0.001	0.148*	0.519*	–	< 0.001
GVII–garlic extract @Fe-MOFs (f)	10	1115	21.8	A	b	c	d	e	
				< 0.001	< 0.001	< 0.001	0.002	< 0.001	–

*P* values: < 0.05 = significant, < 0.001 = highly significant, * = non-significant

#### Brain Cyst Reduction Rate

As illustrated in (Table 4), the mean cyst counts, and their corresponding reduction rates were compared to the infected control group. The garlic extract@Fe-MOFs group (GVII) demonstrated the greatest reduction in cyst burden, achieving a 59.45% decrease. This was followed by the spiramycin@Fe-MOFs group (GV), with a 39.63% reduction, and garlic extract alone group, which showed a 35.27% decrease.

**Table 4:** Mean brain cyst reduction rates within treatment group

Animal groups	Number of mice/groups	Mean brain cyst count	Reduction rate (%)
GI–Healthy control	5	–	–
GII–Infected control	10	2750	–
GIII–Fe-MOFs	10	2170	21.09
GIV–Spiramycin	10	2080	24.36
GV–Spiramycin @Fe-MOFs	10	1660	39.63
GVI–Garlic extract	10	1780	35.27
GVII–Garlic extract @ Fe-MOFs	10	1115	59.45

### Histopathological Changes Findings

#### Brain Sections

**Fig. 4:**
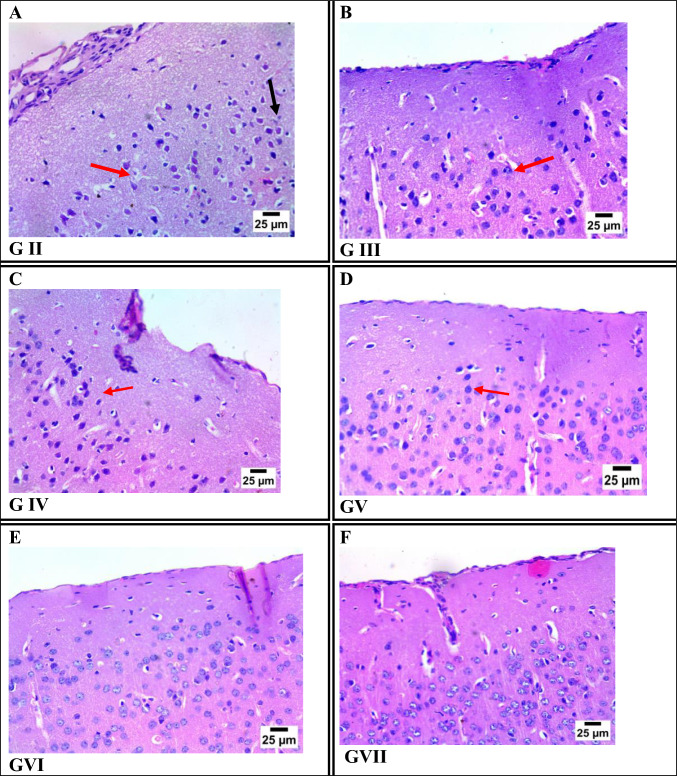
Photomicrograph of brain tissue sections from cerebral cortex stained by H&E representing: **A** GII (infected non-treated), **B** GIII (infected treated by Fe-MOFs), **C** GIV (infected treated by spiramycin), **D** GV (infected treated by spiramycin@Fe-MOFs), **E** GVI (infected treated by garlic extract), **F** GVII (infected treated by garlic Extract@FeMOFs),

Histopathological examination of brain tissue revealed varying degrees of neuronal damage among *T. gondii*-infected groups. The infected control group (Group II) exhibited extensive neuronal necrosis and severe nuclear pyknosis. Groups III (Fe-MOFs) and IV (spiramycin) showed similar levels of neuronal damage, characterized by widespread nuclear pyknosis. Group V (spiramycin@Fe-MOFs) displayed reduced neuronal damage, suggesting partial neuroprotection. Notably, Groups VI (garlic extract) and VII (garlic extract@Fe-MOFs) exhibited minimal to no pathological changes, indicating the most effective neuroprotective response among all treated groups (Fig. 4).

#### Eye Sections

**Fig. 5:**
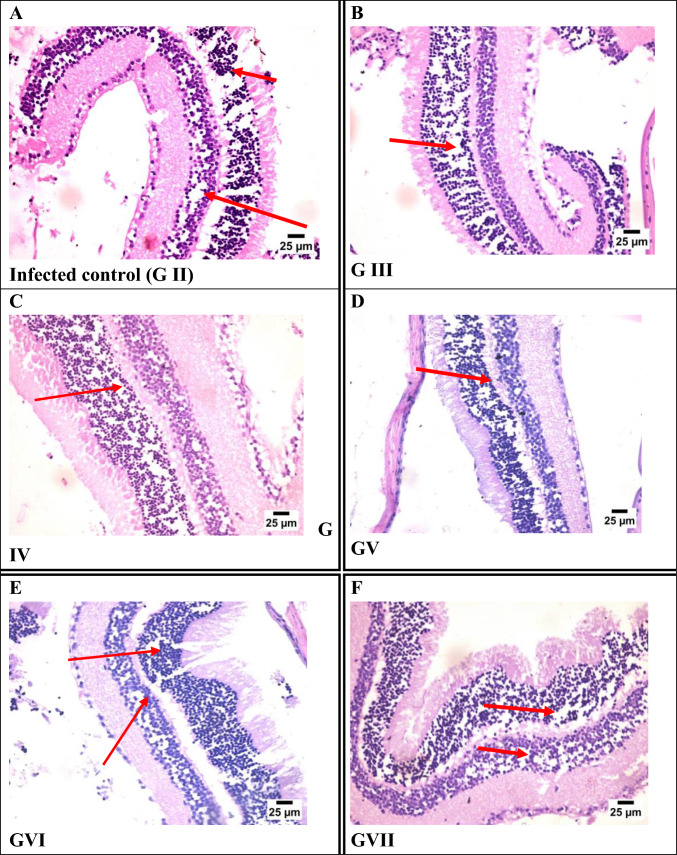
Photomicrograph of eye tissue sections stained by H&E representing: **A** GII (infected non-treated), **B** GIII (infected treated by Fe-MOFs), **C** GIV (infected treated by siramycin), **D** GV (infected treated by spiramycin@Fe-MOFs), **E** GVI (infected treated by garlic extract), **F** GVII (infected treated by garlic extract@Fe-MOFs)

Histopathological examination of eye sections revealed variable degrees of retinal damage among *T. gondii*-infected groups. The infected control group (Group II) exhibited severe disorganization of both inner and outer nuclear layers, indicating marked retinal injury. Groups III (Fe-MOFs) and V (spiramycin@Fe-MOFs) showed severe disruption limited to the outer nuclear layer. Group IV (spiramycin) demonstrated moderate disorganization in both nuclear layers. In contrast, Groups VI (garlic extract) and VII (garlic extract@Fe-MOFs) displayed only mild disorganization, suggesting the strongest protective effect on retinal architecture among the treated groups (Fig. 5).

#### Liver Sections

**Fig. 6:**
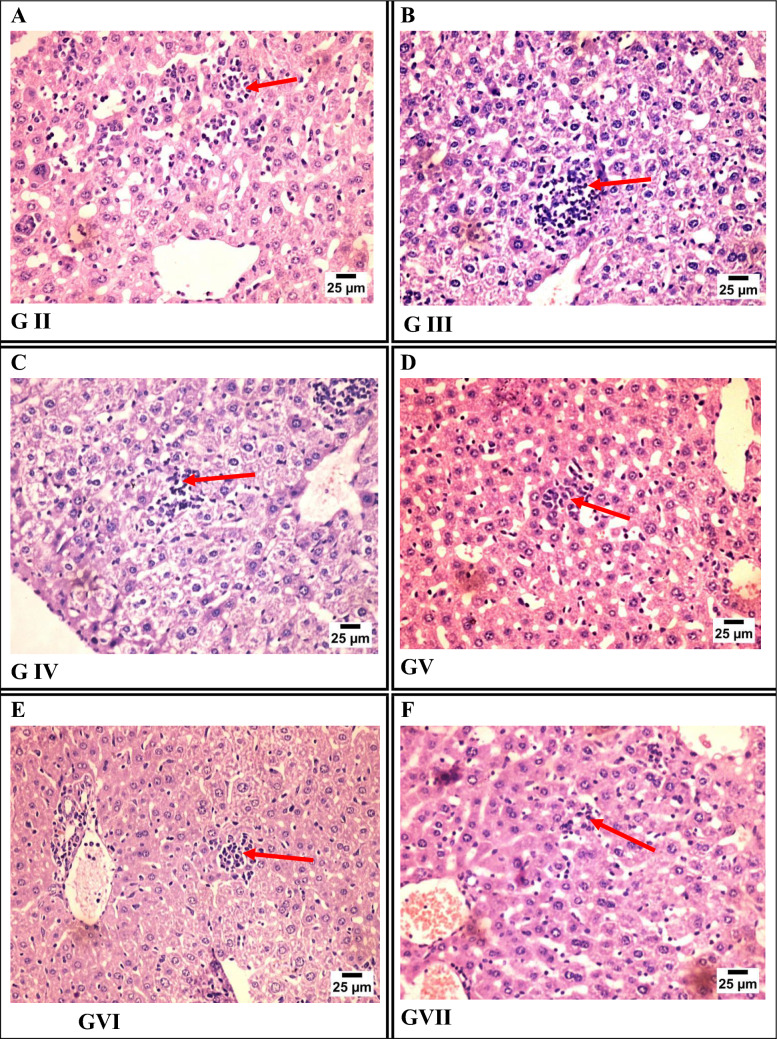
Photomicrograph of liver tissue sections stained by H&E representing: **A** GII (infected non-treated), **B** GIII (infected treated by Fe-MOFs), **C** GIV (infected treated by spiramycin), **D** GV (infected treated by spiramycin@Fe-MOFs), **E** GVI (infected treated by garlic extract), **F** GVII (infected treated by garlic extract@Fe-MOFs)

Histopathological examination of liver sections revealed varying degrees of *T. gondii* cyst burden and inflammatory response among the experimental groups. The infected control group (Group II) showed prominent aggregates of cysts between hepatocytes. Group III (Fe-MOFs) displayed similar cyst aggregation, accompanied by mild mononuclear inflammatory cell infiltration. Groups IV (spiramycin) and V (spiramycin@Fe-MOFs) exhibited a moderate number of cyst aggregates. In contrast, Groups VI (garlic extract) and VII (garlic extract@Fe-MOFs) showed only a mild number of cysts with mild inflammatory infiltration, suggesting a significant reduction in parasitic burden and hepatic inflammation, and indicating a protective effect of garlic-based treatments (Fig. 6).

#### Spleen Sections

**Fig. 7:**
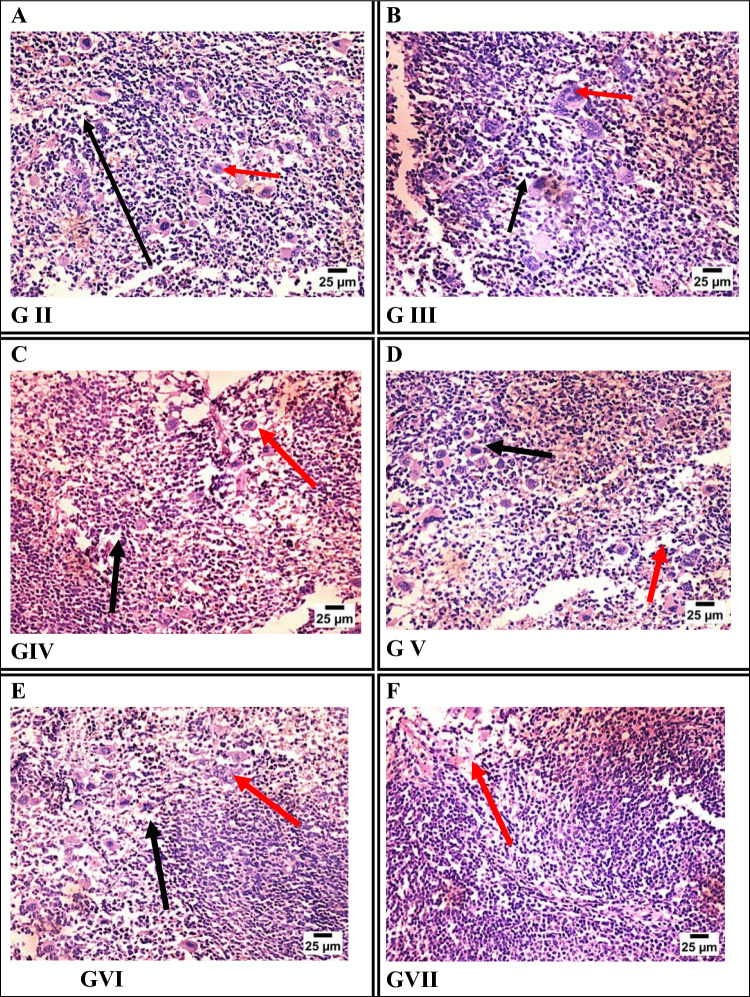
Photomicrograph of spleen tissue sections stained by H&E representing: **A** GII (infected non-treated), **B** GIII (infected treated by Fe-MOFs), **C** GIV (infected treated by spiramycin), **D** GV (infected treated by spiramycin@Fe-MOFs), **E** GVI (infected treated by garlic extract), **F** GVII (infected treated by garlic extract@Fe-MOFs)

Histopathological examination of spleen sections demonstrated varying degrees of white pulp depletion and immune disruption among *T. gondii*-infected groups. The infected control group (Group II) and Group III (Fe-MOFs) exhibited severe white pulp depletion and the presence of megakaryocytes, indicating marked splenic immune impairment. Groups IV (spiramycin), V (spiramycin@Fe-MOFs), and VI (garlic extract) showed moderate white pulp depletion with observable megakaryocytes. Notably, Group VII (garlic extract@Fe-MOFs) displayed only mild white pulp depletion, suggesting preservation of splenic architecture and improved immune response (Fig. 7).

#### Kidney Sections

**Fig. 8:**
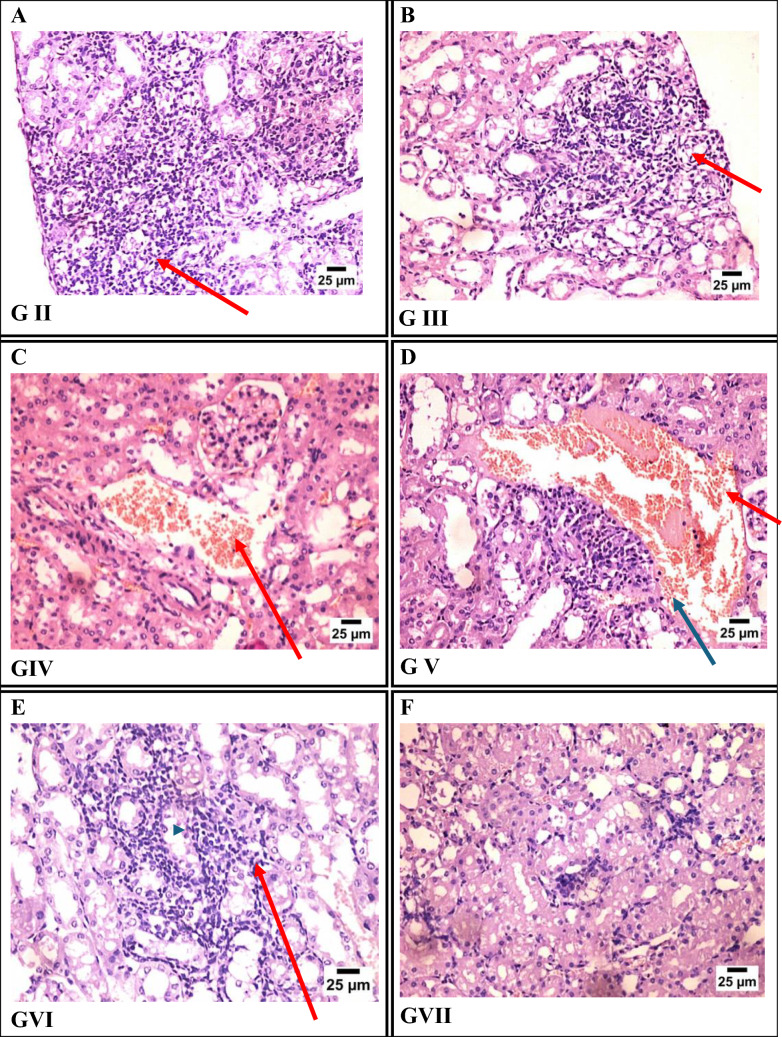
Photomicrograph of kidney tissue sections stained by H&E representing: **A** GII (infected non-treated), **B** GIII (infected treated by Fe-MOFs), **C** GIV (infected treated by spiramycin), **D** GV (infected treated by spiramycin@Fe-MOFs), **E** GVI (infected treated by grlic extract), **F** GVII (infected treated by garlic extract@Fe-MOFs)

Histopathological examination of kidney tissues revealed varying degrees of inflammation among T. gondii-infected groups. The infected control group (Group II) showed severe renal inflammation, characterized by dense infiltration of mononuclear inflammatory cells between renal tubules. Group III (Fe-MOFs) and Group VI (garlic extract) exhibited moderate inflammatory cell infiltration. Group IV (spiramycin) and Group V (spiramycin@Fe-MOFs) demonstrated congestion of peritubular blood vessels, with Group IV showing minimal inflammatory infiltration and Group V showing mild infiltration, suggesting partial renal protection. Notably, Group VII (garlic extract@Fe-MOFs) displayed only mild inflammatory cell infiltration, indicating the most effective protection against renal damage among all treated groups (Fig. 8).

### Molecular Results

Table 5 showed that the mean values of CT and the mean DNA concentrations of *Toxoplasma* P 29 gene (cyst) after treatment in all experimental groups expect GI which has no CT or *Toxoplasma* infection. All experimental groups give a positive result with clear variations in the product quantities and a statistically significant difference between control group GII and others infected treated groups.

**Table 5:** Mean CT values and concentrations of the cysts in the brain tissues of experimental groups

Groups	Sample number	CT value	Concentration (µg/µL)	Significance	*P* value
G1 (non-infected, non-treated)		No CT	None	–	–
GII (infected, non-treated)	1	20.89	0.2262	–	–
	2	17.12	0.3371		
	3	18.05	0.3258		
	4	19.21	0.2439		
	5	19.00	0.2286		
GIII	6	30.03	0.1411	Significant	< 0.05
	7	32.51	0.0950		
	8	32.63	0.1872		
	9	30.02	0.2021		
	10	35.56	0.2040		
GIV	11	23.31	0.1263	Significant	< 0.05
	12	21.16	0.1951		
	13	23.26	0.1089		
	14	22.74	0.1760		
	15	22.48	0.1838		
GV	16	35.37	0.0870	Significant	< 0.05
	17	33.13	0.0976		
	18	35.82	0.1335		
	19	35.26	0.0883		
	20	36.28	0.1639		
GVI	21	26.32	0.1566	Significant	< 0.05
	22	28.91	0.1013		
	23	28.20	0.1197		
	24	26.30	0.1100		
	25	26.01	0.1094		
GVII	26	22.75	0.1313	Significant	< 0.05
	27	23.89	0.0100		
	28	25.68	0.0497		
	29	23.37	0.0541		
	30	23.62	0.0536		

## Discussion

Toxoplasmosis is certainly a significant public health problem [1]. The drugs used to treat toxoplasmosis often have drawbacks such as toxicity, occurrence of relapse and the development of drug resistance. Therefore, the search for new effective with low toxicity drug is required [12]. *Allium sativum* (garlic) is a natural plant that has an inhibitory effect on a variety of parasitic diseases [17, 18]. MIL-101-NH2 is a popular iron-based metal-organic framework (Fe-MOF) used in medicine [26, 27].

This study evaluates the therapeutic potential of garlic extract@Fe-MOF nanoparticles in a murine model of chronic toxoplasmosis, hypothesizing that the nano-formulation will improve drug delivery and reduce toxicity compared to free garlic extract.

The vitro bioassay showed that garlic extract, and Fe-MOFs alone were cytotoxic to normal human cells at higher concentrations, with 95% cell mortality at 100 µg/ml. In contrast, garlic extract@Fe-MOFs showed no cytotoxicity and increased cell growth by 44.7%, indicating enhanced biocompatibility. These results suggest that nano formulation with Fe-MOFs improves the safety and therapeutic potential of garlic extract. Similar results were reported by Fattahi et al. [26] and Gedikoglu [27]

This study showed a mild to moderate reduction in brain parasite cyst burden in all treated groups chronically infected with the *T. gondii* ME49 strain, compared to the untreated group, which had the highest cyst count (2750 ± 29.4).

The group treated by Fe-MOFs alone showed the lowest efficacy, achieving a cysts reduction response rate of only 21.09% compared to the other treated groups. GabAllah et al. [45] reported that the administration of metal nanoparticles, such as silver and gold, showed therapeutic potential in the treatment of toxoplasmosis.

This effect can be attributed to the properties of Fe-MOFs, which enhance immunity against infection. Also, Fe-MOFs can adsorb microorganisms, damage cell membranes, inhibit enzyme function and induce oxidative stress, leading to microbial death and thereby limiting the spread and multiplication of pathogenic organisms [46, 47]. Therefore, Fe-MOFs have been documented as antimicrobial agents effective against a wide range of pathogenic microorganisms.

Our findings indicate that garlic extract, with a reduction brain cysts response rate of 35.27%, was more effective than spiramycin at 24.36%, suggesting that garlic extract has a notable anti-toxoplasmic effect. Dardona et al. [18] reported that garlic shows promise as a natural treatment for toxoplasmosis and other parasitic infections by reducing pro-inflammatory cytokine levels. Also, Naggar et al. [37] reported that spiramycin alone reduced brain cysts by 42.4%, accompanied by mild to moderate tissue damage reduction in mice with chronic toxoplasmosis.

Garlic extract@Fe-MOFs reduced brain cysts by 59.45%, while spiramycin@Fe-MOFs achieved a 39.63% reduction in infected mice. These findings suggest that combination therapy is more effective than monotherapy in treating chronic toxoplasmosis, likely due to a synergistic effect. Similar results were reported by Hagras et al. [48] and Abdel-Wahab et al. [49], who found that nanoparticle-loaded combination therapies more effectively reduced parasitic burden compared to single-agent treatments.

Similarly, a study conducted by El-Shafey et al. [41] found that curcumin@MOFs significantly reduced brain cysts in *T. gondii* ME49-infected rats compared to MOFs alone, demonstrating the enhanced efficacy of the curcumin-loaded formulation.

Although both spiramycin@Fe-MOFs and garlic extract@Fe-MOFs showed therapeutic potential, garlic extract@Fe-MOFs demonstrated superior efficacy. This may be due to the Fe-MOFs enhancing garlic extract’s stability, altering its hydrophilicity, enabling targeted delivery, and controlling its release, thereby improving uptake and therapeutic impact on infected tissues.

Nevertheless, none of these regimens were able to completely elimination of the mice brain cysts. This finding is also in agreement with the studies by El Naggar et al. [37], and Heidari et al. [50], who reported that current anti-*Toxoplasma* medications are unable to fully eradicate tissue cysts from the host.

In the present study, a histopathological examination of tissue sections from the brain, eye, liver, spleen, and kidney of chronically infected mice with *Toxoplasma* revealed a pronounced therapeutic effect of garlic treatment, either administered alone or loaded on Fe-MOF. This intervention led to reduction in tissue inflammation, cellular degeneration and fibrosis. These findings are consistent with previous reports demonstrating the antiparasitic efficacy of garlic and metal–organic frameworks (MOFs).

The molecular method for identification and quantity of parasitic cyst loads in infected tissues spacemen are considered effectiveness. Because in our study we found the highest parasitic cysts which showed in infected untreated group (G2) while the lowest cystic load seen in group treated with garlic extract@Fe-MOFs. This result confirmed the histopathological and parasitological findings.

Collectively, our results demonstrate that loading garlic extract onto Fe-MOFs resulted in the formation of a novel compound with a highly biocompatible formulation, showing therapeutic potential. This combination not only enhanced the biological activity of garlic extract but also benefited from the efficient drug delivery properties of Fe-MOFs. Therefore, the garlic extract@Fe-MOFs combination therapy appears to be a promising candidate for the treatment of chronic toxoplasmosis.

## Recommendation

We need further studies to be done to assess the optimal doses of garlic extract and mechanism of action of this formulation. Also, we need further studies to know which specific components of garlic are released from garlic extract@Fe-MOFs and study the cytotoxicity may differ in other normal cell types.

## Data Availability

The datasets used and/or analyzed during the current study are available from the corresponding author on reasonable request.
